# Strip-loaded Mach–Zehnder interferometer for absolute refractive index sensing

**DOI:** 10.1038/s41598-024-53326-3

**Published:** 2024-02-06

**Authors:** Isaac Doughan, Kehinde Oyemakinwa, Olli Ovaskainen, Matthieu Roussey

**Affiliations:** Center for Photonics Sciences, Department of Physics and Mathematics, University of Eastern Finland, 80101 Joensuu, Finland

**Keywords:** Integrated optics, Optical sensors

## Abstract

This paper presents the design, fabrication, and characterization of a Mach–Zehnder interferometer (MZI) on a strip-loaded platform specifically developed for the measurement of refractive index variations in liquids. A novel approach of large opening in the sensing region enhances the sensitivity by using the analyte as the loading strip. This allows surpassing the performances of the conventional perforations and trenched-based MZIs and prevent protecting the reference arm. The implementation of this design resulted in a high confinement factor of approximately 23$\%$ in the cladding, enabling an effective interaction between the evanescent field and analyte. Ethanol-water solutions with varying concentrations were used as analyte for the characterization of the device. The strip-loaded waveguide was a 200 nm TiO_2_ thin film on a silicon dioxide wafer and a 1.2 $\upmu$m wide for 800 nm-thick nLOF resist served as loading strip. The sensing area featured a 10 $\upmu$m wide open-ing in the loading material with a sensing length of 5 mm. The homogeneous sensitivity was experimentally determined to be 0.128, demonstrating the effectiveness of the proposed design, which enabled a refractive index change monitoring of 10^-6^.

## Introduction

**Figure 1:**
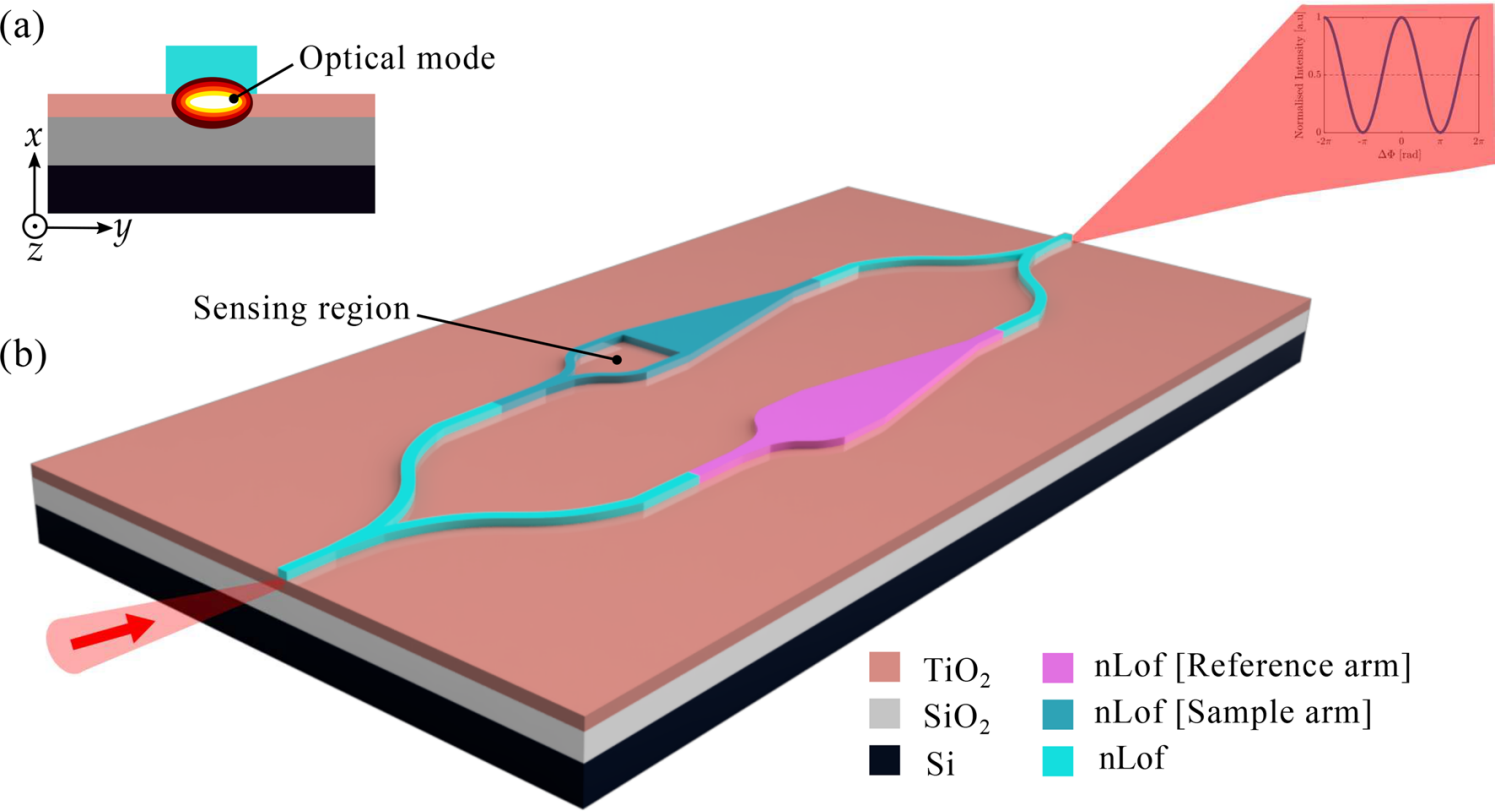
Strip-loaded Mach–Zehnder interferometer configuration. (**a**) Cross section geometry of the strip-loaded waveguide showing the region of mode propagation confinement. The substrate is an oxidized silicon wafer. The guiding layer is TiO_2_ thin film with thickness of 200 nm. The strip atop is AZ-2070 - nLOF polymer. The dimensions of the polymer strip are 1200 nm wide and 800 nm high. (**b**). A 3D drawing of the strip-loaded Mach–Zehnder interferometer. Both the reference and sample arm are made of the same nLOF polymer. A large opening is performed in the sample arm to let the analyte in contact with the titania film. The reference arm is not influenced by the refractive index surrounding the MZI.

Mach–Zehnder interferometers (MZIs) offer numerous applications in several fields including for instance sensing^1,2^, telecommunications^3,4^, and quantum computing^5,6^. However, the design and integration of Mach–Zehnder interferometer sensors in integrated photonics platforms remain challenging, particularly with strip-loaded platforms^7^. A strip-loaded waveguide consists of a thin film of high refractive index confining vertically light and a strip patterned atop this slab waveguide allowing the channeling (2D confinement) of light^8^. This configuration creates a waveguide structure that guides and confines light along the strip region due to a change in the effective index of the slab waveguide in the region under the strip^9^. Strip-loaded waveguides offer advantages such as effective modal field confinement^10,11^ and low loss^12,13^. It means the mode is buried and nearly independent from the surrounding media. This represents the challenge to overcome to make such a waveguide efficient for evanescent sensing applications. One of the challenges arises from the insufficient presence of light in the cladding for interactions with the analyte. Regular channel MZIs address this challenge by utilizing the analyte as the cladding^14,15^ for strong effective index or phase modulation. It is not applicable to strip-loaded waveguides, where light is only weakly guided in the low-index strip atop the guiding layer, which serves to induce a local effective index change on the slab mode. The strip thickness improves light confinement within the guiding core but simultaneously reduces the light field in the cladding, which is crucial for evanescent field sensing. A direct transfer of concept from one platform to another is therefore not possible and the overall light path has to be rethought compared to classical channel photonic circuits in order to increase the performance of the device. In the realm of strip-loaded Mach–Zehnder interferometers, the prevailing method employs perforations and trenches, such as variously sized circles or diagonally placed ellipses^16^. However, these designs often lead to interferometers with reduced sensitivity, particularly for biochemical sensing, due to the difficulties in surface functionalization caused by the small size of the openings, which restricts liquid infiltration. This paper introduces an enhanced strip-loaded Mach–Zehnder interferometer design, distinguished by a substantial opening in its sensing arm, to measure refractive index variations in liquids and potentially gases. This innovation substantially boosts the interferometer’s sensitivity by employing the analyte as the strip material, enhancing light-matter interaction within a precisely controlled segment of the sensor’s arm, while the rest of the device remains largely unaffected by environmental changes. This design offers two main advantages: firstly, opening the low-index channel exposes the evanescent tail of the mode propagating in the slab, leading to an enhanced light and analyte interaction and an increased sensitivity. Secondly, it provides a larger surface area for surface functionalization, making it suitable for developing ultra-sensitive biochemical sensors with specific functionalization of the surface, in the future. Figure 1a is the schematic of cross section of the proposed strip-loaded waveguide. The substrate is an oxidized silicon wafer with 3 $\upmu$m thick silicon dioxide (SiO_2_). The guiding layer is titania (TiO_2_) thin film with $n_{\text {TiO}_{2}}$ =2.24 at $\lambda$ =1550 nm created using atomic layer deposition (ALD).

The strip, enabling the channeling of the guided mode propagating in the TiO_2_ slab, is made of a polymer resist AZ-2070 (nLOF) with refractive index $n_{\text {nLOF}}$ = 1.601 at $\lambda$ = 1550 nm^17^. Figure 1b shows the 3D sketch of the strip-loaded MZI configuration proposed and studied in this paper. The entire circuit is made of electron beam resist. It includes both the reference and the sample arms as well as the input and output channel waveguides allowing to inject and collect light. It is worth notifying that since nearly no light escapes above the loading strip, there is no need for a protection layer above it, i.e., the analyte can cover the entire sample without disturbing the measurement.

## Materials and methods

### Designing tools

Simulations leading to the design parameters of the Mach–Zehnder interferometer were carried out using the Finite Difference Time Domain (FDTD) method^18^ through the commercial software Ansys Optics 2023 R1.2 suite. The choice of FDTD is because of the importance of precise design analysis. Moreso, Mach–Zehnder interferometers (MZIs) employed in our design leverage the interaction of optical waves over defined path lengths. This underscores the significance of employing time-domain simulations, a domain for which FDTD is quite notable. The adoption of FDTD, therefore, aligns with our objective of critically analyzing the behavior of optical signals within the MZI structure.

To ensure accurate and efficient simulations, the boundary conditions of the simulation region were defined as a perfectly matched layer (PML)^19,20^, which are effectively absorbing scattered fields and unwanted reflections from the boundaries. A mesh size of 20 nm in x-, y-, and z-directions was chosen to achieve numerical convergence of the results while limiting the required computer power. The central illumination wavelength used in the simulations was set at $\lambda$ = 1550 nm. The wavelength is chosen because of the low absorption by water in the short wavelength infrared range. Given the high computational demands and memory requirements for 3D-FDTD simulations, sections of the sensor, such as the splitter and the sensing region, were primarily simulated by 2D-FDTD using the effective index approximation^21^. This method helped to reduce computational time without compromising the accuracy of the results. Note that the effective index approximation is valid since the effective index contrast of the chosen waveguide is low, c.a., 0.1 refractive index unit. The data analysis was facilitated by using MATLAB. Mach–Zehnder Interferometer. The Mach–Zehnder interferometer (MZI) consists, here, of two Y-junctions placed head to tail and linked together. One is acting as a 50/50 power splitter separating the input light evenly between the two arms of the interferometer. The second one recombines the two signals and feeds the output waveguide. Their combination forms the two arms of the interferometer both having the same external geometry. One arm serves as a reference and no opening of the loading strip is made. The other arm includes a large opening, detailed later in this paper, used to perform the sensing. The difference in refractive index between the reference and sample arms leads to a modulation of the output intensity, which depends on the optical path difference between the two arms and, therefore, only the refractive index of the analyte, which is the only varying parameter. The optical path must be extremely well defined in an interferometer with the Y-branch playing a crucial role as it sets the contrast between the reference and the sensing arm. Various types of optical splitters, including Y-branch splitters with varying complexities, have been employed to distribute optical signals across multiple outputs, serving diverse applications ranging from 1$\times$2 to 1$\times$128^22–27^ configurations. A notable challenge with Y-branch splitters lies in achieving a balanced splitting ratio in the arms, as asymmetry can lead to an uneven distribution of power in the output waveguide. However, it has been demonstrated that these minor non-uniformities do not significantly impact the splitting characteristics of Y-branch splitters, particularly for configurations below 1$\times$16^28^. In the context of this paper, a 1$\times$2 splitter is sufficient. The standard predefined shapes for Y-branches are typically the S-bend arc and the S-bend sine. In the design of our 1$\times$2 Y-branch splitter, we utilized the Bézier curve to define the geometric structure of our Y- branch, which is described by the equation^29^ :

1 $$\begin{aligned} B(t) = \sum _{i = 0}^{n} b_{i,n}(t)B_{i} \end{aligned}$$

Here, $B_i$ represents the control points of the curve and the Bernstein polynomial of degree *n*, denoted as $b_{i,n}(t)$, is expressed as follows:

2 $$\begin{aligned} b_{i,n}(t) = \left( \frac{n}{i} \right) t^i(1-t)^{n-1}, i = 0,1,2,\ldots ,n \end{aligned}$$

Compared to other predefined shapes, the Bézier curve offers more degrees of freedom by adjusting the control points and the degree of the Bézier curve in the Ansys Optics design environment. The design of the sensing arm involves opening the nLOF strip formed by two nLOF walls. It resembles a Y-branch with no guiding in the branches but only in the gap between them. It acts then as a taper enlarging the mode in the TiO_2_ slab waveguide to form a larger sensing zone, while preventing losses due to leakage, since light remains trapped in the slab. When the gap between the two walls in the sensing region is far larger than the wavelength of the propagating mode, one can approximate the sensing region as a slab waveguide consisting of SiO_2_ as the substrate, TiO_2_ as the guiding layer and air as the cladding. The air cladding an open window for the analyte, i.e., the ethanol-water solution, application on the sensor. The reference arm has the same geometry as the sample arm except that no opening area is performed on the nLOF of strip, which acts as the reference material for the MZI sensor. The end of the sensing region consists of a tapper designed to collect and converge light from the sensing region making it guide under the nLOF strip again. The signals from the sensing arm and reference arm are coupled back through the combiner. The modes interfere with each other. The interference I of the two signals is given as^30,31^

3 $$\begin{aligned} I = I_S + I_R + 2\sqrt{(I_SI_R)} cos(\Delta \phi ) \end{aligned}$$

where $I_S$ and $I_R$ are the intensities of the sensing and the reference arm, respectively. $\Delta \phi$ is the phase difference between the reference and sensing signals, which is due to the difference of refractive index between the analyte and the reference material, i.e., nLOF. The refractive index of the analyte depends directly on the concentration of ethanol in water, which is our targeted measure. During the characterization process, the MZI’s output is measured in terms of power. The output power of the MZI is given as

4 $$\begin{aligned} P_{out} = P_{in} cos^2(0.5\Delta \phi ) \end{aligned}$$

where $P_{out}$ and $P_{in}$ are the output and input power of the MZI, respectively. By varying the refractive index of the analyte and measuring the output power, the phase change $\Delta \phi$ can be calculated. The phase change between the reference arm and the sensing arm is described by^32^

5 $$\begin{aligned} \Delta \phi = \frac{2\pi }{\lambda } L \Delta n_{\text {eff}} \end{aligned}$$

where L is the interaction length, $\lambda$ is the wavelength of light in vacuum and $\Delta n_{\text {eff}}$ is the difference in effective refractive index between the guiding modes. From Eq. 5, one can deduce that long sensing areas ensure high sensitivity even for very small refractive index variations in the sample arm. This makes MZI a very powerful tool to detect minute changes of the refractive index. Sensitivity refers to how much the sensor’s output changes in response to a change in the refractive index of the surrounding medium (analyte). It is a measure of the responsiveness of the sensor to changes in the parameter being measured. In the proposed strip-loaded MZI, the analyte is deposited on the whole sensing region making the sensitivity of the device homogeneous. The homogeneous sensitivity S_H_ of the MZI is calculated as^33^

6 $$\begin{aligned} S_H = \frac{\delta n_{\text {eff}}}{\delta n_{\text {c}}} \end{aligned}$$

which represents how the effective index of the propagating mode changes with the refractive index of the analyte in the cladding ($n_c$). The homogeneous sensitivity of the device obtained experimentally is simply the slope of the linear fitting for the calculated values of $\Delta n_{\text {eff}}$ versus $\Delta n_{\text {c}}$ relating to the varying concentrations of the ethanol-water solutions.

### Fabrication techniques

**Figure 2:**
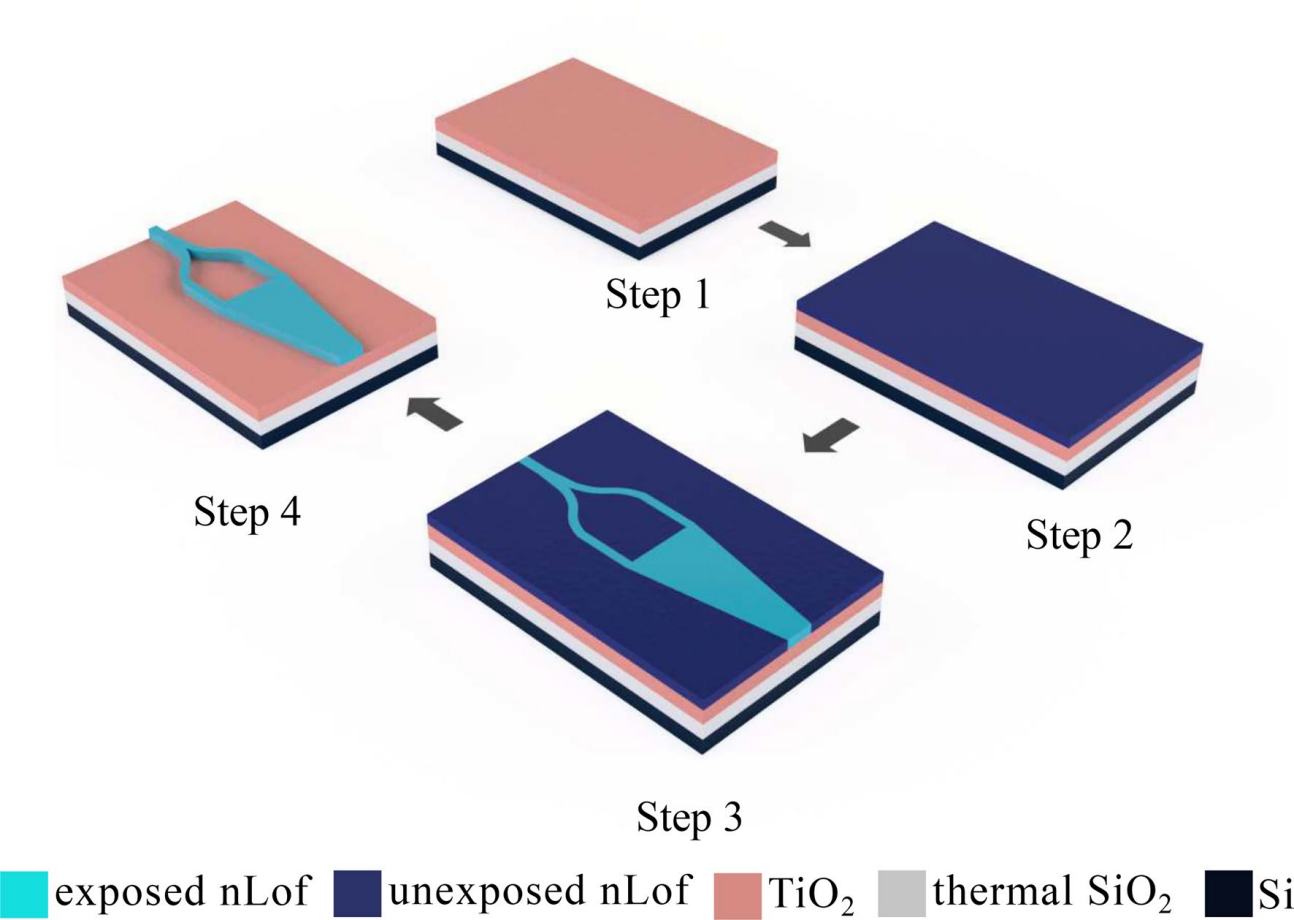
Fabrication process flow. Step1: ALD coating of the substrate with a 200 nm TiO_2_. Step 2: spin coating of the nLOF resist. Step 3: electron beam lithography. Step 4: development of nLOF, cleaning of the wafer, and cleaving of the chips prior to characterization.

3” oxidized silicon wafers (3 $\upmu$m of thermal SiO_2_, Si-Mat, Kaufering, Germany) are used as substrates. An O_2_ plasma (PE-50 plasma cleaner, Plasma Etch) is performed to remove impurities at the surface of the wafer. The process flow is depicted in Fig. 2.

The required 200 nm thick TiO_2_ film was formed by ALD (Beneq TFS200) using a thermal process with TiCl_4_ and O2 as precursors (2857 cycles) at 120^∘^C to ensure an amorphous material. After the ALD process, the sample was treated again with O2 plasma and spin-coated with the resist AZ 2070 nLOF diluted with AZ EBR (2:3). 800 $\upmu$l of the dilution was pipetted and spin-coated at 1500 rpm for 60 s. The sample was soft baked on a hot plate at 110 ^∘^C for 60 s. A coating thickness of 799 nm was measured afterwards with a profilometer (Dektak 150). The sample was exposed with electron beam lithography (EBL) system (Raith EPBG5000+HR). Doses of 40 and 60 $\upmu$C/cm^2^ were used in the exposure. The sample was post baked at 110 ^∘^C for 60 s and developed in AR 300-47 (Allresist GmbH) for 90 s, stopped in DI water for 30 s and rinsed under DI water before being dried with N2 blow. Finally, the several chips on the wafer were manually cleaved to be ready for characterization.

### Analyte sample preparation

Three Ethanol-water solutions were prepared with mass concentrations of c_1_ = 0.00178 $\%$ (w/w), c_2_ = 0.0167$\%$ (w/w), and c_3_ = 0.178$\%$ (w/w)^34^. The required amounts of ethanol (solute) and deionized water (solvent) were measured accurately using a precise Sartorius Entris analytical balance (ENTRIS224i-1S/0.1mg). For each solution, a specified mass of ethanol was diluted with an appropriate amount of deionized water to achieve a total mass of 100 g. A thorough mixing ensured the homogeneity of the solutions. The refractive index of the solvent at $\lambda$ = 1550 nm was taken as 1.3154 and the refractive index of ethanol as 1.3520 at the same wavelength^35^. The refractive index change of the ethanol-water solutions increases with the ethanol concentration and is calculated using the formula $\Delta n_{\text {c}}$ = 0.0006 $\times$ C_eth_, where $\Delta n_{\text {c}}$ represents the refractive index change and C_eth_ is the mass concentration of ethanol^36^.

### Characterization methods

**Figure 3:**
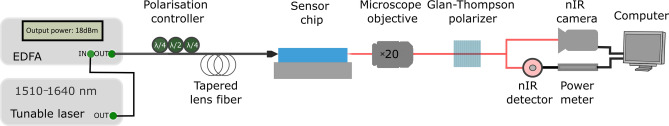
Schematics of the sensor characterization setup. The detector end can be switched from power meter to camera.

The strip-loaded MZI was characterized using the setup configuration shown in Fig. 3^37^. A power meter is connected to a computer for the recording of the measured power. The end-fire coupling method was used to inject and collect light. Light from a tunable laser source (Tunics plus), set at $\lambda$ = 1550 nm, is coupled into the input of the sensor using a tapered lens fiber (OZ Optics). The output power is measured and recorded using a power meter with near infrared detector (Thorlabs PM100D with S122c detector) and stored on a computer. The desired polarization (TE) of the input light was set with the help of a polarization controller and verified with a Glan-Thompson GTH10M polarizer from Thorlabs. A microscope objective (Olympus Plan N, N.A. = 0.4, 20$\times$) was used to image the output mode on the detector of the power meter. Alternatively, to the detector, the mode can be observed using Point Grey Chameleon CMLN-13S2M camera, allowing a verification, at any time of the experiment, a well-coupling to the waveguide without any modification of the measurement system. The power values were recorded for various concentrations of the ethanol-water solution. Ideally, one would consider the use of a fluidic cell, but the nature of our sensor does not necessarily require it, since the reference arm is not dependent on the outer medium. Experimental procedure. To ensure precision and avoid contamination, we carefully transferred samples from each prepared solution into separate containers, using pipettes dedicated to each solution. The MZI chip was mounted onto a translational stage using custom-built 3D printed sample holders. To illuminate the sensor, a TE light source (TE/TM polarization fractions of 99.7$\%$) was injected into the input of the MZI. The power measurement using the software was started. The output with air as cladding was recorded and used as the baseline for all measurements. With the help of a pipette (FINNPIPETTE), we deposited 20 $\upmu$l of water on the sensing region and the output power was recorded. With the help of a balloon end pipette, water was sucked up of the chip and the power measurement was also observed till the power value stabilized to the baseline. The process was repeated for all the solutions. All the results were recorded in one file for further analysis under MATLAB.

## Results and discussion

Optimization of the waveguide parameters. When selecting the waveguide parameters for refractive index sensing applications, an essential figure of merit is the confinement factor of light in the cladding. This factor determines how much the optical mode extends outside the slab waveguide, which dictates first the light/analyte interaction in the opening area and second, the no-interaction with the very external medium when the nLOF strip is present. Additionally, it is crucial to ensure that the waveguide supports only the fundamental quasi-TE mode for enhanced signal strength. Moreover, the variation of effective index between the regions with analyte and with nLOF resist is also linked to the confinement factor, which then affects the modulation of the output signal. Therefore, the geometry of the waveguide is optimized to meet these requirements.

### Modal effective index

**Figure 4:**
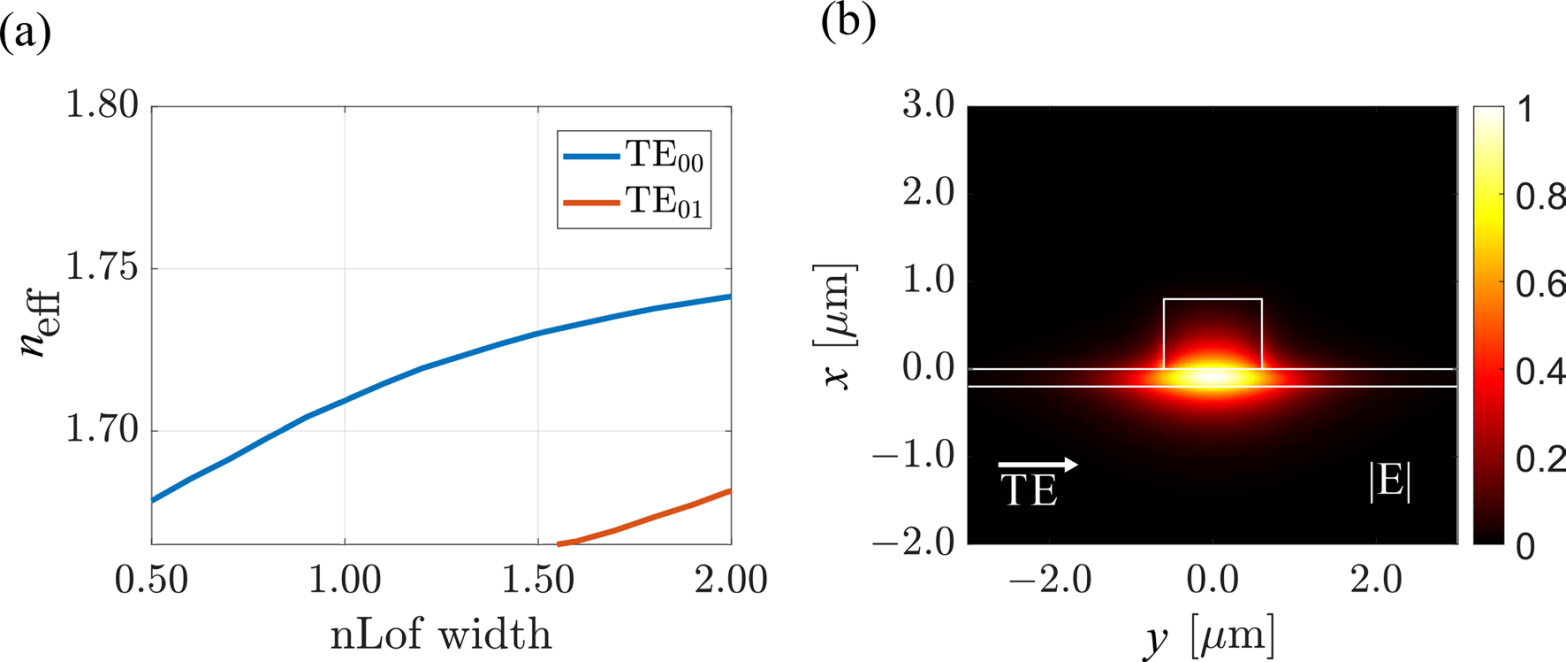
Waveguide optimization. (**a**) Effective index of the strip-loaded waveguide as a function of the nLOF strip width for the fundamental TE_00_ and the first order TE_01_ mode. The thicknesses of both TiO_2_ and nLOF polymer are kept constant at 200 nm and 800 nm, respectively. (**b**) TE_00_ mode distributions with nLOF width of 1200 nm.

The waveguide configuration used in this work has initially been studied through nano-imprint replication technique. The results showed propagation losses for the fundamental mode in TiO_2_ slab waveguide of about 1 dB/cm using prism coupler measurement, which makes it a suitable waveguide for this work. We have studied through FDTD simulations the effective index variation of the fundamental TE_00_ mode and the first order TE_01_ mode. The refractive indices considered for the materials have been measured using prism coupler (Metricon) and are at $\lambda$ = 1550 nm: $n_{\text {TiO}_{2}}$ =2.2452, for titanium dioxide deposited by ALD. $n_{\text {SiO}_{2}}$ =1.444, for the silicon oxide layer on the silicon wafer substrate. $n_{\text {nLOF}}$ =1.5991, for the strip material. To ensure proper operation of the MZI, the evanescent tail of the guided mode should not interact with the medium above the loading-strip in the non-sensing regions. It means that the thickness of nLOF should be large enough. For this reason, the thickness of the nLOF strip was chosen to be 800 nm. Figure 4a shows the variation of the effective index of the fundamental quasi-TE mode as a function of the nLOF width for a fixed thickness of TiO_2_ and nLOF of 200 nm and 800 nm, respectively. It is obvious from Fig. 4a that, below a strip width of 1500 nm, only the fundamental TE_00_ mode is sustained and guided in the waveguide.

**Figure 5:**
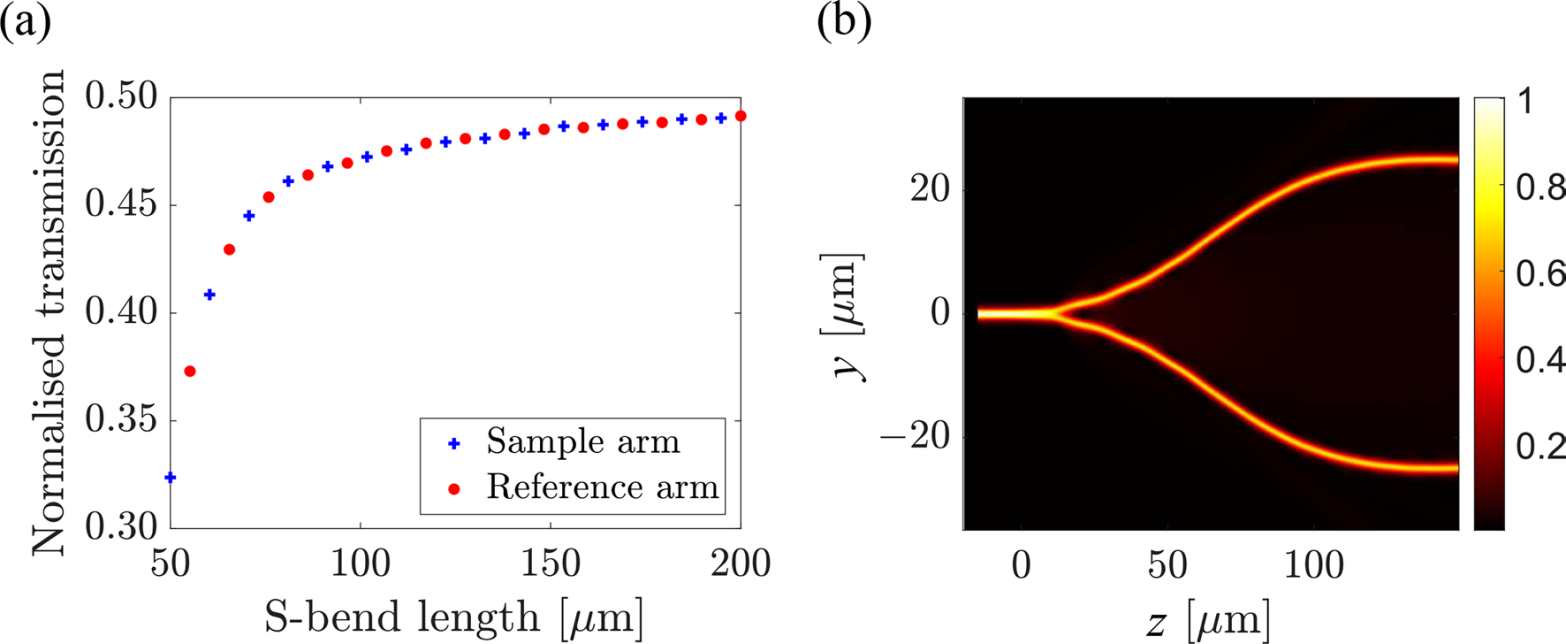
(**a**) Normalized transmittance at Y-branch splitter outputs as a function of the S-bend length for both the sample arm (plus blue curve) and reference arm (dotted red curve). (**b**) Electric field distribution along the Y-branch splitter with an S-bend length of 150 $\upmu$m. The gap between the two arms is 50 $\upmu$m.

It can also be observed that within the nLOF width of interest, effective index of both the fundamental and first order TE modes increases with an increase in the nLOF width. For this reason, we chose the width of the nLOF strip to be 1200 nm. This ensures good confinement of the mode with weak amount of light on the side of the strip and also considers the fabrication tolerances. Figure 4b shows the electric field profile of the fundamental mode solved using the Finite Difference Element (FDE) mode solver in Ansys Optics mode. The choice of appropriate geometry for a waveguide for evanescent sensing goes beyond considering the effective index only. One must also consider the confinement factor of the electric field in the cladding of the waveguide. The confinement factor in the cladding is important as it determines the amount of light that interacts with the analyte. This plays a key role in determining the sensitivity of the sensor. The relationship between the effective index and the confinement factor is inversely proportional. This means that as the effective index of the waveguide increases, the confinement factor (in the cladding) reduces in general. It implies that, the stronger the field in the cladding region for an efficient analyte and light interaction, the lower the confinement of light in the TiO_2_ guiding layer, which is usually synonym of higher propagation losses. One must make a trade off between the effective index and the confinement factor to obtain waveguide structure satisfying both a high sensitivity and low propagation losses. For the chosen parameters, we obtain a confinement factor in the analyte of $\Gamma _{\text {Analyte}}$ $\approx$ 23$\%$. This will be explained further in this section. Mach–Zehnder interferometer. Here, we discuss the main results in the development of the Y-branch splitter and the sensing area which are the most important parts of the strip-loaded MZI. The Y-branch splitter. Figure 5a shows the normalized transmittance at the output of the splitter as a function of the S-bend length of the splitting part.

At this stage, the sample and the reference arm are similar. Beyond an S-bend length of 150 $\upmu$m, the transmittance changes insignificantly. Figure 5b shows the electric field profile along the Y-branch splitter. The length of the S-bend is 150 $\upmu$m and the gap between the two arms is 50 $\upmu$m. One can observe from the field profile that the input beam splits evenly between the two branches and remains guided without leaking in the slab. The sensing area. The sensing area of the proposed MZI employs a wide opening of the nLOF strip turning the sensor area into a 3-layer asymmetric slab waveguide with the air space as the cladding where the analyte will be further deposited.

**Figure 6:**
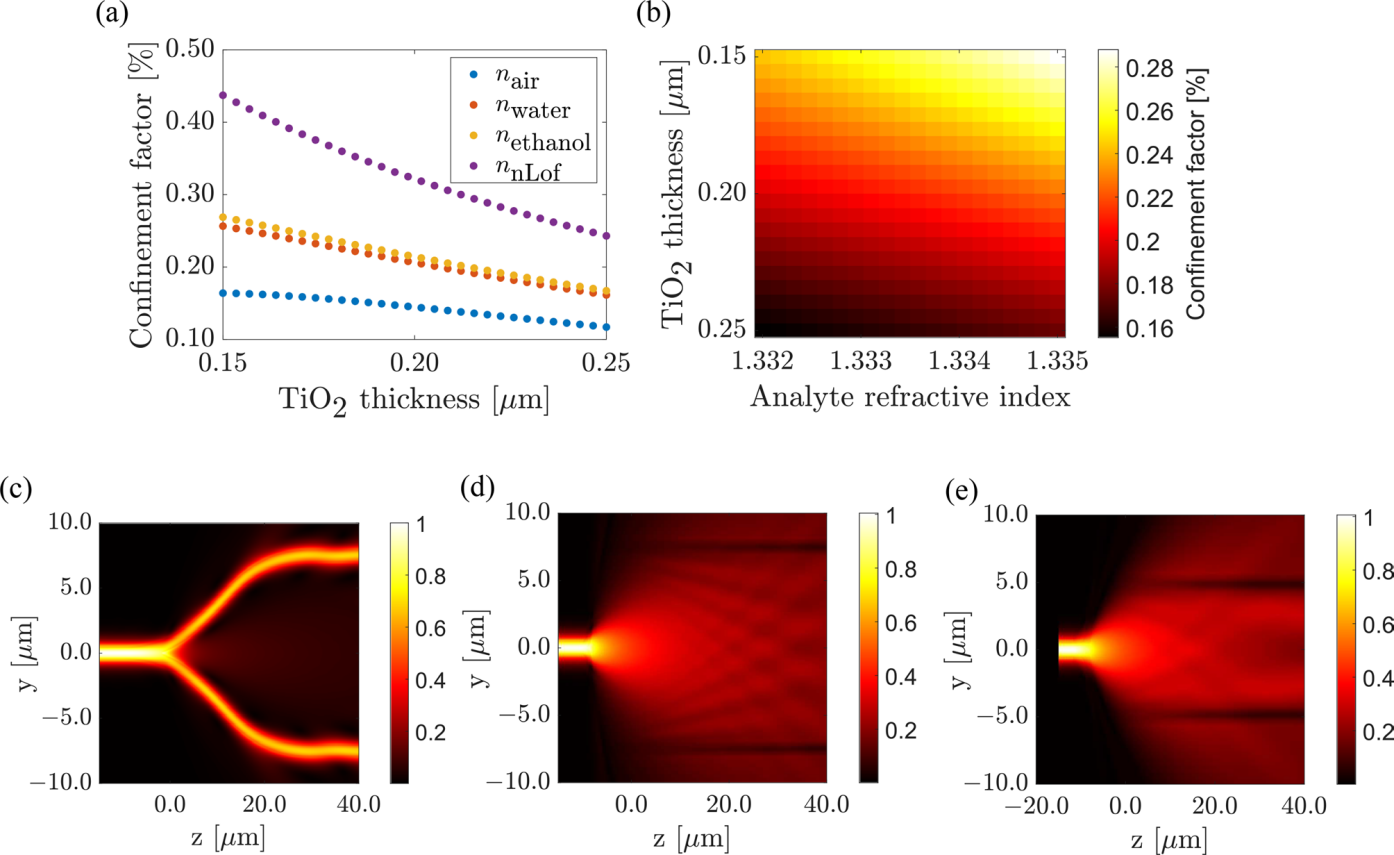
Sensing region simulations (**a**) Confinement factor analysis for different analytes at varying TiO_2_ thickness in the sensing region (**b**) Influence of the TiO_2_ thickness and the refractive index of interest on the confinement factor in the sensing region. Electric field distribution at the input of the sensing region (top view) for S-bend length of (**c**) 40 $\upmu$m and (**d**) 5 $\upmu$m, with 15 $\upmu$m gap between the arms. (**e**) Corresponds to a bend of 5 $\upmu$m and a gap of 10 $\upmu$m.

This technique is possible for a strip-loaded waveguide since the TiO_2_ core region ensures the vertical trapping of light. The modulation of the effective index in the sensing region is greatly influenced by the portion of electric field confined in the cladding (analyte). Figure 6a,b shows the confinement factor as a function of both the TiO_2_ thickness and the analyte refractive index. A relatively high confinement of light of about 23$\%$ in the analyte can be obtained. The confinement factor, a fraction of the optical energy confined within the sensor’s interaction region, is especially crucial because it plays a pivotal role in influencing the sensor’s performance. A high confinement factor as in our case not only ensures a considerable amount of optical energy interaction but also extends the interaction length ensuring minute changes in refractive index or analyte concentration are detected. This prolonged interaction contributes to improved resolution, heightened signal-to-noise ratio, and ultimately enhances overall device efficiency of our MZI.

It can also be seen that the confinement factor is inversely proportional to the thickness of TiO_2_, as expected in view of effective index trend, for a fixed refractive index of analyte. Aiming at a high confinement factor in the analyte, for higher sensitivity, the mode will be poorly confined, and the losses will be high and vice versa. Improving the confinement of the mode in the TiO_2_ also means low confinement factor in the analyte hence low sensitivity. This means that there is a trade off to be made between the sensitivity and losses when designing evanescent field sensors. The sensing region is similar to a Y-branch but light must not be guided by them. Its design is thus performed using the opposite figure of merit than for the splitters forming the MZI. The objective is to lose the propagation along the S-bend so that the signal can propagate straight and experience the refractive index influence of the analyte. The results from the sensing area with water as cladding material are shown in Fig. 6c–e. Figure 6c,d show the electric field distribution simulated for an S-bend length of 40 $\upmu$m and 5 $\upmu$m, respectively. From Fig. 6c, the beam splits and remains guided by the loading strip despite some weak leaking in the slab. On the opposite, from Fig. 6d, the incoming beam is propagating mainly in the open area, ignoring the tight bend and further waveguides. The two arms in the sensing region have the same geometry. The portion of light propagating in the TiO_2_ core in the sensing region exhibits a diverging and lateral expansion behavior which causes some light leakage. This can be mitigated by adjusting the width of the sensing area: the smaller the better. One can also note some interference pattern, similar to Talbot effect, coming from the reflection of the mode on the side channel waveguides. This effect is however negligible due to the low effective index contrast and can be further reduced by reducing the gap between the arms to 10 $\upmu$m, as shown in Fig. 6e. Note that the width of the sensing area is dependent upon the output tapering part at the end of the sensing region. The purpose of the taper is to collect and guide the signals from the sensing region and direct it back to the strip-loaded waveguide. The low index contrast of the waveguide requires that the taper section becomes long as the light modulation inside the guiding layer is small. Consequently, the length of the designed taper was set to 2 mm. This is to ensure that all possible light coming from the sensing region is collected and guided for a nearly adiabatic operation.

### Fabricated sample

**Figure 7:**
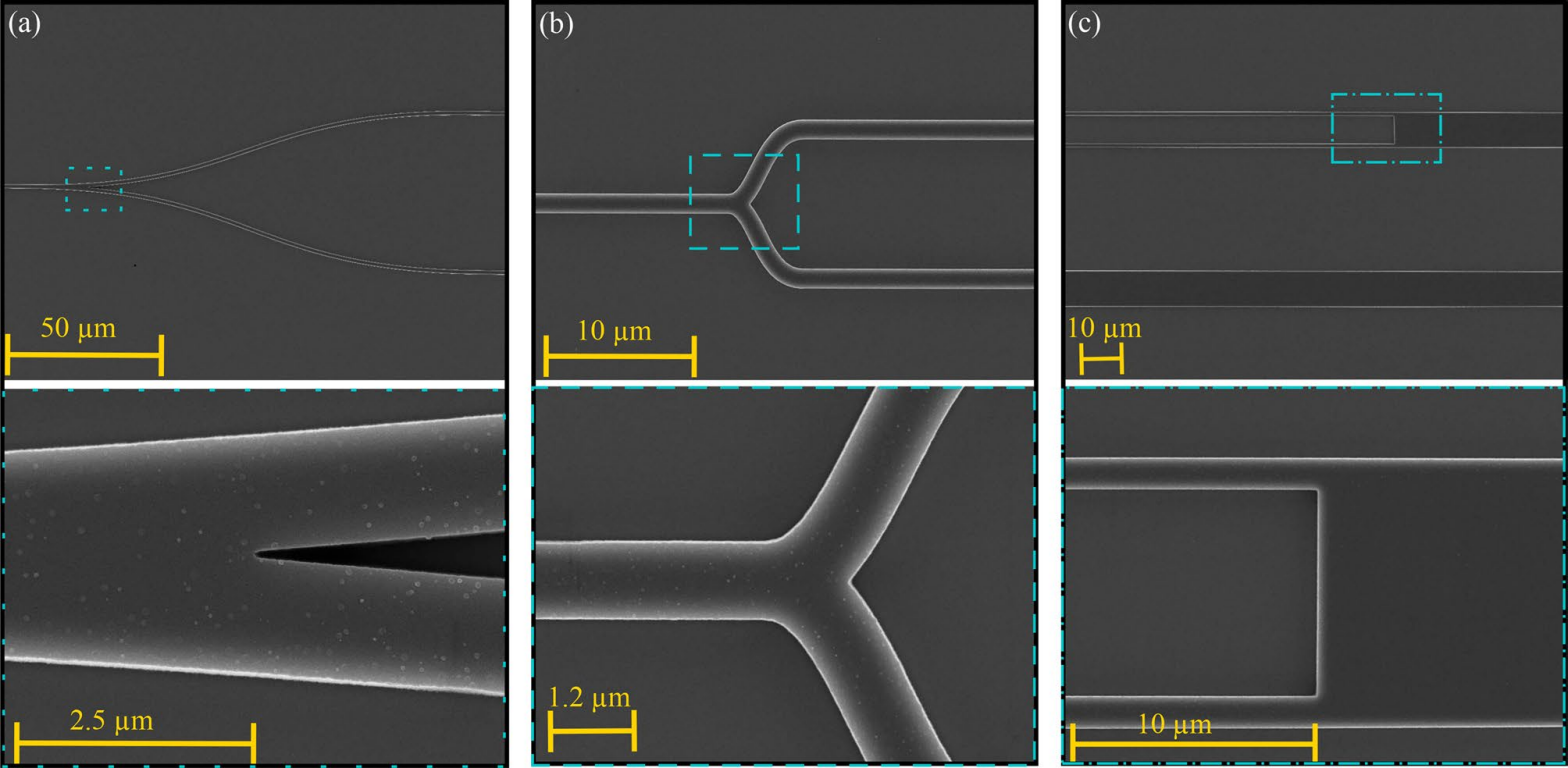
Scanning electron microscope pictures (top views) of different sections of the Mach–Zehnder interferometer. (**a**) Y-branch splitter with a zoomed in on the separation of the two arms. (**b**) Input of the sensing region showing part of the sample arm. (**c**) Latter part of the sensing region and beginning of the taper section.

Figure 7 compiles the scanning electron microscopy (SEM) pictures of the main parts of the fabricated MZI. Figure 7a shows the Y-branch splitter of the MZI with a long S-bend. Figure 7b shows the input of the sensing region with a very short S-bend. Figure 7c shows the end of the sensing region and the beginning of the taper section. Refer to Fig. 1b for the definition of the different sections of the MZI. In Fig. 7c, one can see the two arms, one with an opening and the other without.

**Figure 8:**
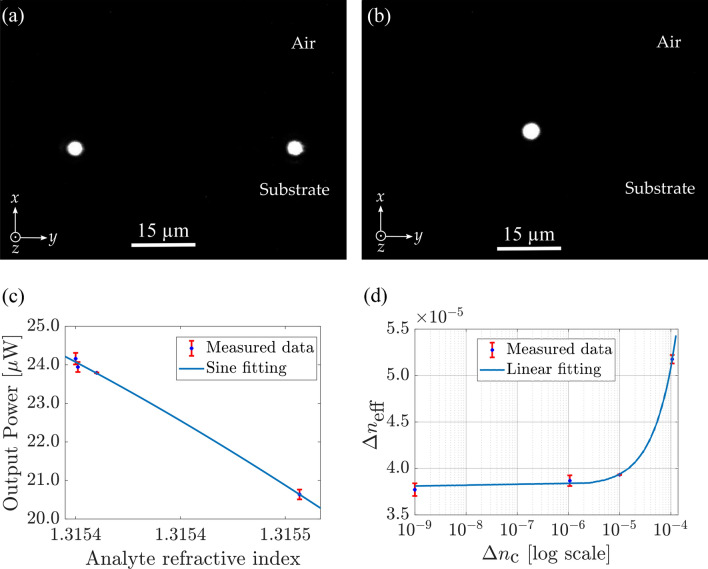
Characterisation results. (**a**) Photograph of the TE fundamental mode at the output of the Y-branch splitter with S-bend length of 150 $\upmu$m. The separation between the two arms is 50 $\upmu$m. (**b**) Photograph of the TE fundamental mode at the output of the strip-loaded Mach–Zehnder interferometer with air as cladding material in the sensing region. (**c**) Measured output power as a function of the analyte refractive index with sine fitting. (**d**) Calculated effective index change as a function of refractive index change of the analyte with fitting in microns. The sensing length is 5 mm. The slope represents a homogeneous sensitivity of 0.128.

### Experimental characterization

A specific sample was fabricated to study the splitters used in the construction of the MZI. Figure 8a shows the output fundamental mode of the Y-branch splitter. As can be seen the input beam splits nicely and the Y-branch splitter works effectively well. This has been verified by measuring the intensity at the output of both waveguides. Figure 8b shows the output mode image of the MZI with air as the cladding material in the sensing region. The reference arm is isolated from any external perturbation and the variation in the sensing arm is only due to the change in concentration of the ethanol-water solution. The measured output power obtained from the power meter is plotted as a function of the analyte refractive index in Fig. 8c. The output power follows a sinusoidal curve as predicted by the theory and such function was used to fit the experimental data. It is to be noted that the length of the sensing zone is a means of setting an offset in terms of path length difference. It has been chosen so that the range of refractive index corresponds to the maximal variation (highest slope in the sine function) of the interference response. The maximum estimated output power when light in the two arms is in phase is about 30 $\upmu$W. It can also be seen from Fig. 8c that the output power at the output of the MZI interferometer decreases with an increase in refractive index within the range of our analyte which is in line with our simulations. According to the theory ruling the MZI, it shows that the data points are the highest slope of the sine function allowing for the highest sensitivity, as expected. Figure 8d shows a plot of the effective index change as a function of refractive change of the ethanol-water concentration. Note that the data is represented on a semi logarithmic scale to render better the large range of refractive index. The correlation coefficient of the fit is $\rho$ = 0.9987, which shows an excellent validity for the model and proves that only the refractive index change of the analyte affects the sensing results. The slope of the fitting curve determined by Eq. 6 gives a homogeneous sensitivity of $S_H$ = 0.128, which is excellent according to the state-of-the-art^38^. The operating range in terms of concentration of the studied MZI is of several orders of magnitude ranging from 0.00178 $\%$ (w/w) to 0.178 $\%$ (w/w) and corresponding to a smallest refractive index change of the analyte of 1.10^-6^. This is limited by the uncertainty on the measurements that can be greatly reduced by pig-tailing the chip device and building a fluidic cell to ensure an easier application of the solution on the sensing area. Moreover, the range of refractive index, i.e., the range of possible measurable concentration can be tuned by adding an offset to the reference arm. This can be done very accurately by changing slightly the effective index of its fundamental mode, e.g., by varying the width of the loading strip locally, or for a bigger offset, by changing its length. This counts among the advantages of using a strip-loaded waveguide as a platform for sensing.

## Conclusion and outlook

A strip-loaded Mach–Zehnder interferometer with a wide opening in the sensing arm has been demonstrated where the variation in the sensing region is detected by measuring the changes in the output power. The MZI features a wide opening in the sensing arm which yields a confinement factor of 23$\%$. The fabrication techniques employed resulted in fine structures allowing interferometry at the most effective response due to the high control of the dimensions. The analyte used was varying concentration of ethanol-water solutions. The homogeneous sensitivity of the sensor was experimentally determined to be 0.128. We demonstrated a minimal variation of refractive index of 1.10^-6^. for a concentration variation of 0.00178$\%$ (w/w). Looking ahead, the development of our strip-loaded Mach–Zehnder interferometer sets the stage for advancing sensor technologies. The precision it offers and its immunity to environmental medium variations due to the buried mode (except for the sensing area) form a key-building block for the design smart sensors using strip-loaded platforms. Integrating this interferometer with neural networks holds the promise of enhancing sensor capabilities, enabling real-time analysis, and providing adaptive responses to dynamic environmental changes. This synergy could usher in a transformative era of intelligent sensor systems across diverse applications.

## Data Availability

The datasets generated during and/or analysed during the current study are available from the corresponding author on reasonable request.
